# A kinematic model for 3-D head-free gaze-shifts

**DOI:** 10.3389/fncom.2015.00072

**Published:** 2015-06-10

**Authors:** Mehdi Daemi, J. Douglas Crawford

**Affiliations:** ^1^Department of Biology and Neuroscience Graduate Diploma, York UniversityToronto, ON, Canada; ^2^Centre for Vision Research, York UniversityToronto, ON, Canada; ^3^CAN-ACT NSERC CREATE ProgramToronto, ON, Canada; ^4^Canadian Action and Perception NetworkToronto, ON, Canada; ^5^Department of Psychology, York UniversityToronto, ON, Canada; ^6^School of Kinesiology and Health Sciences, York UniversityToronto, ON, Canada; ^7^Brain in Action NSERC CREATE/DFG IRTG ProgramCanada/Germany

**Keywords:** gaze-shift, saccade, vestibulo-ocular reflex (VOR), head movement, Listing's law

## Abstract

Rotations of the line of sight are mainly implemented by coordinated motion of the eyes and head. Here, we propose a model for the kinematics of three-dimensional (3-D) head-unrestrained gaze-shifts. The model was designed to account for major principles in the known behavior, such as gaze accuracy, spatiotemporal coordination of saccades with vestibulo-ocular reflex (VOR), relative eye and head contributions, the non-commutativity of rotations, and Listing's and Fick constraints for the eyes and head, respectively. The internal design of the model was inspired by known and hypothesized elements of gaze control physiology. Inputs included retinocentric location of the visual target and internal representations of initial 3-D eye and head orientation, whereas outputs were 3-D displacements of eye relative to the head and head relative to shoulder. Internal transformations decomposed the 2-D gaze command into 3-D eye and head commands with the use of three coordinated circuits: (1) a saccade generator, (2) a head rotation generator, (3) a VOR predictor. Simulations illustrate that the model can implement: (1) the correct 3-D reference frame transformations to generate accurate gaze shifts (despite variability in other parameters), (2) the experimentally verified constraints on static eye and head orientations during fixation, and (3) the experimentally observed 3-D trajectories of eye and head motion during gaze-shifts. We then use this model to simulate how 2-D eye-head coordination strategies interact with 3-D constraints to influence 3-D orientations of the eye-in-space, and the implications of this for spatial vision.

## Introduction

Gaze-shifts, i.e., rapid reorientations of the line of sight, are the primary motor mechanism for re-directing foveal vision and attention in humans and other primates (Bizzi et al., 1971; Tomlinson and Bahra, 1986a; Tomlinson, 1990; Guitton, 1992; Corneil and Munoz, 1996). Natural gaze-shifts in most mammals incorporate the complex coordination of eye-head movements including a saccade toward the target, a more sluggish head movement and usually the vestibulo-ocular reflex (VOR) which keeps the eye on target during the latter parts of the head motion (Tomlinson and Bahra, 1986b; Guitton et al., 1990; Freedman and Sparks, 1997; Roy and Cullen, 1998). These components have been modeled with considerable success by several authors (Robinson, 1973; Jurgens et al., 1981; Galiana and Guitton, 1992), but the three-dimensional (3-D) aspects of gaze control have only been modeled once (Tweed, 1997), and some more recently discovered properties of this system have never been addressed.

In the current study, we incorporate recent experimental findings into a new model for three-dimensional (3-D) gaze control, verify our mathematical approach with the use of simulations, and then use the model to explore some poorly understood aspects of eye-head coordination. In particular, we explore the interactions between the spatiotemporal rules of eye-head coordination, the 3-D constraints on eye/head orientation, and the resulting orientations of the eye (and thus retina) in space. These interactions are crucial both for understanding gaze motor coordination, and for understanding its visual consequences. Before addressing such interactions, we need to consider the basic kinematics of the eye-head gaze control system, progressing from one dimensional (1-D) to 3-D aspects.

### Overview of gaze kinematics

In one dimension, gaze control kinematics reduces to the amplitude and temporal sequencing of eye and head motion (Tomlinson and Bahra, 1986b; Guitton and Volle, 1987; Guitton, 1992; Sparks et al., 2002). The typical sequence of events includes a saccade, followed by a slower head movement and a compensatory vestibulo-ocular eye movement. The aspects of this progression that we will explore here include the variable timing of saccade, head movement, and VOR, the influence of initial eye and head orientations, relative magnitudes of the contribution of these different phases to the gaze-shift, and where the head falls in space after the gaze-shift.

Additional complexity emerges when one considers gaze-shifts from a two-dimensional (2-D) perspective. For example, the eye and head provide different relative contributions to horizontal and vertical gaze motion, which must be predictably accounted for saccades to produce accurate gaze shifts (Freedman and Sparks, 1997; Goossens and van Opstal, 1997), and for the eye and head to end up in the right positions after the VOR (Crawford and Guitton, 1997a; Misslisch et al., 1998).

Finally, gaze control reaches its highest degree of complexity in 3-D (Glenn and Vilis, 1992; Freedman, 2001; Crawford et al., 2003a). First, there is an added dimension of motion control: torsion, which roughly corresponds to rotations of the eyes and/or head about an axis parallel to the line of sight pointing directly forward. Torsion influences direction perception for non-foveal targets (Klier and Crawford, 1998), binoclular correspondence for stereo vision (Misslisch et al., 2001; Schreiber et al., 2001), and must be stabilized for useful vision (Crawford and Vilis, 1991; Fetter et al., 1992; Angelaki and Dickman, 2003). More fundamentally, a 3-D description requires one to account for the non-commutative (order-dependent) properties of rotations (Tweed and Vilis, 1987; Hepp, 1994). These non-commutative properties influence not only ocular torsion and the degrees of freedom problem, but also gaze accuracy, for reasons related to reference frame transformations (Crawford and Guitton, 1997b; Crawford et al., 2011).

The location of a visual stimulus is initially described in an eye-centered reference frame by the pattern of light that falls on the retina and the resulting activation of eye-fixed photoreceptors (Westheimer, 1959). Whereas the orientation of the eye and the brainstem premotor commands for eye movement are encoded in a head-centered reference frame (Crawford and Vilis, 1992; Crawford, 1994), head orientation and head movements are encoded in a coordinate system attached to the shoulder (Klier et al., 2007). This is because the eye muscles which move the eyes are fixed to the head and the neck muscles which move the head are fixed to the shoulder (Farshadmanesh et al., 2007). This necessitates reference frame transformations from eye-fixed visual coordinates into head and/or shoulder-fixed motor coordinates (Sparks and Mays, 1990; Klier et al., 2001). These transformations can sometimes be avoided in 1-D and 2-D models of gaze-shifts that borrow the math of the translational motion to approximate rotation, but this approach cannot be followed when the full properties of 3-D rotation are incorporated. In this case, reference frame transformations must be embedded in the fundamental structure of the model in order for the math to work (Tweed, 1997; Crawford and Guitton, 1997b; Blohm and Crawford, 2007; Blohm and Lefevre, 2010). This too will be incorporated into our model.

Another factor to consider is the biological constraints that limit the degrees of freedom of the eye and head orientations to a subset of their mechanically possible range. For example, suppose the eye is described in a fixed coordinate system, and the eye undergoes fixed-axis rotations. An infinite number of rotational axes can be employed to bring the eye from any given initial orientation toward a final 2-D gaze direction, but they will each result in a different amount of final ocular torsion around the line of sight. However, Donders' law states that only one final eye orientation is achieved for each 2-D gaze direction, and thus only one axis of rotation can be used (Glenn and Vilis, 1992; Crawford et al., 2003a). Orientation of the eye relative to the head and orientation of the head relative to the shoulder obey Donders' law between gaze-shifts when the head and body are in normal upright postures (Misslisch et al., 1994; Klier and Crawford, 2003). Orientation of eye-in-head has also been shown to obey the Listing's law (Ferman et al., 1987a,b; Tweed and Vilis, 1990; Straumann et al., 1991); If torsion is defined as rotation about the axis parallel to gaze at the primary eye position, then Listing's law states that eye orientation always falls within a 2-D horizontal-vertical range with zero torsion known as Listing's plane (LP). Note that in order to maintain eye orientation in LP, rotations must occur about axes that tilt out of LP as a function of eye position, a phenomenon known as the half angle rule (Tweed and Vilis, 1990). In contrast, orientation of head-on-shoulder has been shown to obey the Fick strategy (Glenn and Vilis, 1992; Crawford et al., 1999; Klier et al., 2007) where horizontal rotation occurs about a body-fixed vertical axis, vertical rotation occurs about a head-fixed horizontal axis, and the remaining torsional component is held near zero. Mechanical factors appear to aid these constraints by implementing some of the position-dependencies required to deal with non-commutativity. In particular, eye muscles appear to implement the half-angle rule (Demer et al., 2000; Ghasia and Angelaki, 2005; Klier et al., 2006). However, it is clear that mechanical factors do not *constrain* eye and head torsion, because the eye violates Listing's law during the VOR (Misslisch et al., 1998; Crawford et al., 1999; Glasauer, 2007), and the head constraint can be violated voluntarily or when used as the primary mover for gaze (Ceylan et al., 2000).

Note that these systems seem to be primarily concerned with enforcing Donders' law during fixations at the end of the gaze-shift (when both the eye and head are relatively stable) perhaps because of their various implications for sensory perception. Listing's law is also obeyed during saccades with the head-fixed (Ferman et al., 1987b; Tweed and Vilis, 1990). However, when the head is free to move, both the eye (Crawford and Vilis, 1991; Crawford et al., 1999) and head (Ceylan et al., 2000) are known to depart from their Donders' ranges during gaze movement, for reasons that will be described below. This also suggests additional aspects of neural control that, to date, have only been considered for the eye.

Thus, a complete model of the head-free gaze-shifts needs to incorporate both the reference frame transformations and some solution to the behavioral constraints described above. Further, such a model should plan for spatial and temporal coordination of saccade, head movement and VOR. Furthermore, variability of the contribution of head movement to the gaze-shift, the variability of the sizes of saccade and VOR and the variability of these contributions in different spatial directions have to be considered. These factors interact in complex fashions that have only partially been explored. Again, this remains an important topic, because it has fundamental implications for both vision and motor control. But before attempting to address this goal, we will briefly review previous attempts to model gaze control, ranging from early models of the 1-D saccade system to the most recent 3-D model of eye-head coordination.

### Gaze control models: from 1-D saccades to 3-D eye-head control

Attempts to model the gaze control system have generally advanced from 1-D models of head-restrained saccades toward multi-dimensional models of head-unrestrained gaze-shifts. The first models of gaze-shift were dynamic models of one-dimensional head-fixed saccades. Robinson (1973) assumed that saccades are driven by a fast feedback loop allowing trajectory corrections on the fly (Robinson, 1973). Jurgens et al. (1981) observed that despite the variability of the duration and speed of the saccades their accuracy is almost constant, and considered this observation favoring the hypothesis of local feedback (Jurgens et al., 1981). Next, 1-D saccade models were generalized to 2-D (oblique) and 3-D saccades (Freedman and Cecala, 2008). van Gisbergen et al. (1985) observed for oblique saccades that the horizontal and vertical components of the movement start simultaneously and are adjusted relatively such that straight trajectories are produced (van Gisbergen et al., 1985). Then they found that a model based on a common source of motor command for horizontal and vertical components agrees with the data rather than a model based on independent 1-D motor commands for the two components. In parallel to this, many of these principles, combined with models of the VOR, were incorporated into models of eye-head gaze control. For example, Morasso et al. (1973) developed this idea that the head movement during gaze-shift attenuates the saccade amplitude by an amount equal to the VOR (Morasso et al., 1973). Galiana and Guitton (1992) proposed a kinematic model of eye-head coordination in one dimension, in which they introduced the idea of VOR gain changing as a function of gaze-shift amplitude (Galiana and Guitton, 1992).

The development of 3-D models of gaze-shifts followed a similar course, but shifted forward by a decade. Tweed and Vilis (1987) mathematically proved, through non-commutativity of 3-D rotations, that the 3-D saccades should be planned based on 3-D kinematics of the eye rather than linear generalization of the 1-D models (Tweed and Vilis, 1987). Subsequent 3-D models of the saccade generator either focused on the question of eye muscle contribution to Listing's law (Quaia and Optican, 1998; Raphan, 1998), reference frame transformations for saccades (Crawford and Guitton, 1997b), or interactions between saccades and vestibular system (Glasauer et al., 2001; Crawford et al., 2003b, 2011). Tweed (1997) proposed selection of specific final orientations of eye and head by defining constraints on their torsional components (Tweed, 1997). Although he specified Listings' law for eye, he didn't specifically considered Fick constraint for head. Since then, some aspects of Tweed's framework have been used for modeling other aspects of visual-motor integration (Blohm and Crawford, 2007). Several theoretical studies have also developed expanded mathematical descriptions of Listing's and Donders' laws (Ghosh et al., 2014; Hess and Thomassen, 2014) while others have been inspired by gaze physiology to design camera (eye) and its platform (head) movement controllers for robotic applications (Peters and Qureshi, 2010; Mao and Chen, 2012).

To our knowledge, in the past 18 years there has been no further attempt to incorporate such constraints into a model of the eye-head gaze control system. Our aim here was to (1) do this in a systematic fashion with the use of an Engineering Design approach (Pahl et al., 2007), (2) evaluate the resulting model against known properties of the 3-D gaze control system through simulations, and then (3) use further model simulations to explore a topic that has received little attention in the gaze-control community: the sensorimotor implications of interactions between 2-D eye-head coordination strategies and the 3-D constraints underlying Listing's law and the Fick strategy.

## Model formulation

### Overview

In order to understand and model the kinematics of the gaze control system, we have chosen an approach that is usually used in the branch of mechanics called “engineering design” (Pahl et al., 2007). This approach includes three levels: First, static kinematic model, which involves deriving the desired positions and patterns of motion for different components in the plant for meeting a kinematic end. Second, temporal discretization, which involves determining a time-framework and associating specific temporal growth functions to different desired motions and then deriving the velocities and accelerations of different components as functions of time. Finally, the third level involves putting the known kinematic variables, external loads and the mechanical properties of the plant into the equations of conservation of momentum and solving them for the unknown force/torque functions. In this paper, we mainly describe our model at the first level: a static kinematic model for 3-D head-unrestrained gaze-shifts toward visual targets.

**Figure 1:**
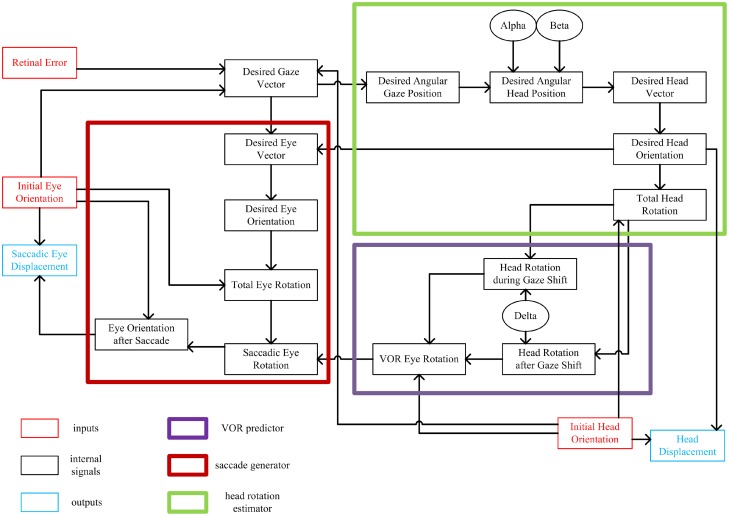
**Flow of information in the static kinematic model**. Red and blue rectangles show model inputs and outputs, respectively. Black ovals are the model parameters. Big thick red box shows the part of the model involved in computation of the saccadic eye movement. Big thick green box shows the part of the model which computes the head movement. Big thick violet box shows the VOR predictor. Each signal is computed from the signals that have inputs to it.

Figure 1 shows a summary of the signals in the model and their relations with each other. The small red and blue boxes are inputs and outputs of the system, respectively. Each signal is mathematically computed from its input signals. The major internal computations can be divided into three categories: one responsible for calculating the total head rotation (large green box), one responsible for predicting the VOR-related eye rotation (large violet box), and one responsible for calculating the saccadic-related eye rotation (large red box). The remainder of this section describes these stages in more detail, and relates them to brain physiology. (The mathematical implementation of these steps is described in the next section.)

This sequence of calculations begins when light is emitted from a target in the periphery onto the retina. This we represent as *retinal error*, the eye-centered 2-D vector which characterizes the distance and direction of the retinal image of the target relative to the fovea. In our model, this is geometrically equivalent to gaze 2-D motor error in retinal coordinates, and thus could represent spatial activity in the brain at any point from the retina to the deep layers of the superior colliculus (Klier et al., 2001; DeSouza et al., 2011). Desired gaze (eye-in-space) vector, a unit vector directing toward the target, is calculated from retinal error and the internal knowledge of the initial 3-D orientations of eye-in-head and head-on-shoulder, which could be derived from proprioceptive signals (Steinbach, 1987; Wang et al., 2007) and/or efference copies from “neural integrators” in the brainstem (Cannon and Robinson, 1987; Crawford et al., 1991; Farshadmanesh et al., 2007). Note that this 2-D gaze vector does not yet specify torsion of the eye in space; it is an intermediate computational stage useful in decomposing retinal error into both eye and head components (see below). Thus, the initial stages of the model are based on experimental observations that early gaze centers specify 2-D direction, with implementation of 3-D eye and head constraints further downstream from the superior colliculus (van Opstal et al., 1991; Klier and Crawford, 2003).

In order to calculate the desired head movement (Figure 1; green box), the desired gaze vector is first converted into angular gaze position, a 2-D version of desired gaze vector in spherical coordinates. Desired angular gaze position is then calculated. Desired angular head position is computed from the desired angular gaze position and two model parameters: α and β. These two parameters have been defined to determine where the horizontal and vertical components of head position fall relative to desired gaze direction (Our model also provides provision for initial head position to influence final head position, but we have not simulated this here.) The 3-D desired head direction vector is computed from the 2-D desired angular head position. Desired head orientation that conforms to the Fick strategy (zero torsion in Fick coordinates) is then calculated from the desired head vector. Knowing the initial and desired head orientations, the total fixed-axis head rotation is calculated, and then converted into a head displacement command (see below for physiological interpretation of this output).

In order to generate a saccade that is correctly coordinated with head movement (Crawford et al., 1999), our model first predicts the VOR eye movement that will occur toward the end of the movement (Figure 1; violet box). Assuming the constancy of the axis of head rotation throughout the gaze shift, the total head rotation is broken down into two parts with the aid of one of the model parameters, δ. This parameter defines two phases of the head rotation; a first phase which contributes to the gaze-shift and a second phase which is canceled out by VOR. Knowing the initial head orientation and the two parts of head rotation, then one can predict the ideal VOR eye movement that would stabilize 3-D gaze orientation during the second phase of the head rotation. This is not the same physiological mechanism as the actual VOR (which is driven by signals from the semicircular canal), but in our simulations to avoid redundancy we model an ideal VOR and use this signal both for the prediction and the actual VOR. In real world conditions this behavior would occur thousands of times each day, and thus provide ample opportunity to train a dynamic neural network to learn the calculations described here. The physiological basis for this hypothetical predictive network could involve the brainstem and cerebellum (Crawford and Guitton, 1997a; Crawford et al., 1999).

The last part of the model is involved in computing the 3-D saccade vector (Figure 1; Red box), meaning a saccade that also includes the torsional components required to offset the oncoming VOR (Crawford et al., 1999). Having computed the desired head orientation and desired gaze vector, we first calculate the desired final 2-D eye direction vector relative to head (after saccade and VOR). We then covert this into desired eye orientation (after the saccade and VOR) to fall in the Listing's plane. Knowing the initial and desired eye orientations, we calculate the total fixed-axis eye rotation. Having computed the total eye rotation and the VOR eye rotation, we can finally calculate the saccadic eye rotation. This rotation not only results in foveation of the target but also compensates for all VOR components in a predictive fashion. This is then converted into the desired final eye orientation after the saccade, and initial eye orientation is subtracted from this to produce desired 3-D eye displacement in Listing's plane coordinates. This command is mathematically appropriate to drive the known 3-D coordinates of premotor oculomotor structures (Crawford and Vilis, 1992; Crawford, 1994), and henceforth derivatives of eye orientation coded within the phasic burst of motoneurons (Ghasia and Angelaki, 2005; Klier et al., 2006; Farshadmanesh et al., 2012a). The torsional component of this displacement command might be generated by the nucleus tegmenti reticularis pontis (van Opstal et al., 1996), eventually leading to activation of the torsional burst neurons. Thus, these parts of the model reflect what might happen in the real brain between the superior colliculus (Klier et al., 2001) and the oculomotor burst neurons (Henn et al., 1991; Crawford and Vilis, 1992; Crawford, 1994).

Very little is known about the mathematical details of brainstem and spinal motor commands for the head, but they appear to follow similar principles to that seen in the oculomotor system (Klier et al., 2007; Farshadmanesh et al., 2012a). Therefore, to model the final output of our head control system we also subtracted initial 3-D head orientation from desired 3-D head orientation to obtain a 3-D dispacement command. Note that for such displacement outputs, it is necessary that any further position-dependences, such as the half-angle rule of eye velocities for Listing's law, are implemented further downstream, likely at the level of muscles (Demer et al., 2000; Ghasia and Angelaki, 2005; Klier et al., 2006; Farshadmanesh et al., 2012a,b).

### Basic mathematical framework

**Figure 2:**
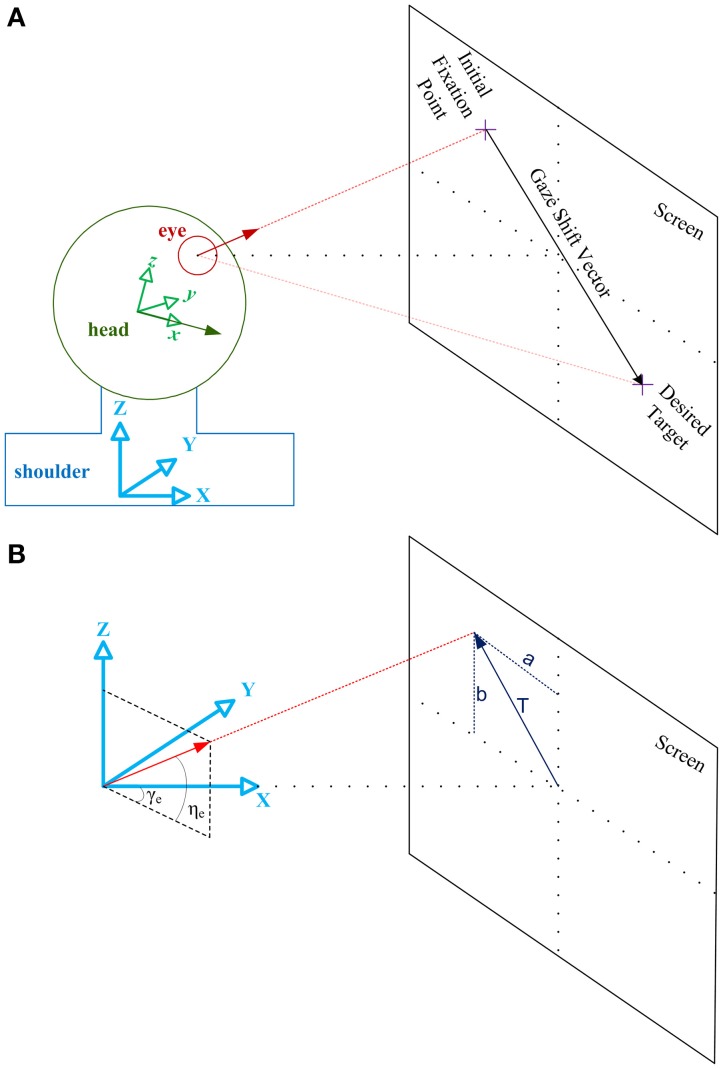
**Illustration of the geometrical framework for studying head-free gaze-shift**. **(A)** Head coordinate system, shown by the green axes fixed to the head, explains everything relative to the head. Shoulder or space coordinate system, shown by the blue axes fixed to the shoulder, explains everything relative to the space. Green vector is the head vector which is fixed to the head and moving with it. Red vector is the eye vector which connects center of the eye ball to the fovea. In the reference condition eye and head vectors are aligned in the same direction and intersect with the center of the screen. Eye vector defined in head coordinate system, $\vec{e}$, is called eye-in-head vector and eye vector defined in space coordinate system, $\vec{g}$, is called gaze vector. Head vector, $\vec{h}$, is defined only relative to space. **(B)** Space coordinate system is drawn again to show how eye vector is characterized in space to represent the gaze vector. Gaze vector, or eye vector in space coordinates, is a unit vector which shows where the eye is fixating. Gaze vector can have a 2-D angular representation based on the angles [η_*e*_, γ_*e*_] it creates in spherical coordinates with the axes (the same applies to the head vector with angles [η_*h*_, γ_*h*_] not shown here). Gaze vector can be derived if we know where on the screen the subject is fixating, which is characterized by vector $\vec{T}$.

As illustrated in Figure 2, *eye vector* (red) is a vector fixed to the eye ball aligned from the center of the eye ball to the fovea. Assuming the head as a sphere, *head vector* (green) is a vector fixed to the head, aligned from the center of this sphere to the nose. Initially, eye vector intersects with the screen at the initial fixation point. Gaze-shift is to be planned to foveate the desired target, i.e., move the eye vector to intersect the desired target location on the screen. This shift of eye vector is executed by a coordinated pattern of eye and head movements.

As illustrated in Figure 2, we define a coordinate system attached to the shoulder and fixed to the space. {*X*, *Y*, *Z*} of this so-called space coordinate system are respectively orthogonal to the coronal, sagittal, and axial anatomical body planes. We also define a coordinate system attached to the head which moves with the movement of the head. We define reference condition as the straight-ahead configuration of eye and head where {*x*, *y*, *z*} of the head coordinate system is aligned with {*X*, *Y*, *Z*} and eye vector is aligned with *x* and *X*. For instance, in a conventional experimental setup for eye movement research, where the subject is sitting in front of a screen, reference condition is typically when the subject is fixating the center of the screen and eye vector and head vector are parallel.

Eye vector is called eye-in-head vector, $\vec{e}$, when defined in head coordinate system and is called gaze vector, $\vec{g}$, when defined in space coordinate system. Head vector, $\vec{h}$, is only defined relative to space coordinate system. For any configuration of oculomotor system, eye-in-head orientation, **E**, head orientation, **H**, and gaze orientation, **G**, are rotation matrices which rotate $\vec{e}$, $\vec{h}$, $\vec{g}$, respectively, from the reference position to their current configuration (letters “*r*,” “*i*,” and “*d*” as subscripts, denote reference, initial and desired conditions):

(1) $$\vec{e}=E\times {\vec{e}}_{r}$$

(2) $$\vec{h}=H\times {\vec{h}}_{r}$$

(3) $$\vec{g}=G\times {\vec{g}}_{r}$$

At any arbitrary configuration, if we rotate the eye-in-head vector by head orientation matrix we will derive the gaze vector. So, gaze orientation is always the multiplication of head and eye-in-head orientations:

(4) G=H×E

We define $\vec{c}$, the 2-D angular gaze position, and $\vec{b}$, the 2-D angular head position, based on the defining angles of the eye and head vectors in the spherical version of the space coordinate system (these angles are shown for eye vector in Figure 2B; the same applies for the head vector.)

(5) $$\vec{c}=\left[\gamma_{e}\;;\;\eta_{e}\right]$$

(6) $$\vec{b}=\left[\gamma_{h}\;;\;\eta_{h}\right]$$

Gaze and head vectors can be directly derived from the spherical angles:

(7) $$\vec{g}=\left[\cos\eta_{e}\;.\;\cos\gamma_{e}\;;\;\cos\eta_{e}\;.\;\sin\gamma_{e}\;;\;\sin\eta_{e}\right]$$

(8) $$\vec{h}=\left[\cos\eta_{h}\;.\;\cos\gamma_{h}\;;\;\cos\eta_{h}\;.\;\sin\gamma_{h}\;;\;\sin\eta_{h}\right]$$

We also define the target position on the screen by the vector $\vec{T}$ = [*a*; *b*] as it is illustrated in Figure 2B. If “*t*” is the distance between the eye and the center of the screen, $\vec{T}$ and the components of $\vec{g}$ can be derived from each other:

(9) $$\vec{g}=\frac{1}{\sqrt{t^{2}+a^{2}+b^{2}}}\left[t\;;\;a\;;\;b\right]$$

(10) $$\vec{T}=t\;\times \;\left[g^{Y}\;;\;g^{Z}\right]$$

The main input of the oculomotor system is supposed to be the retinal error. In our formulation, we define a 3-D version of this signal, $\vec{g}$_*RE*_, as the desired gaze vector relative to the initial gaze orientation:

(11) $$\vec{g_{RE}}\;=\;G_{i}^{-1}\;\times \;\vec{g_{d}}$$

A 2-D angular version of this signal can also be derived from the previous vector:

(12) $$RE\;=\;\left[cos^{-1}(g_{RE}^{Z})\;;\;cos^{-1}\left(\frac{g_{RE}^{Y}}{sin\left(cos^{-1}\left(g_{RE}^{Z}\right)\right)}\right)\right]$$

### Motor mechanisms of eye-head movement

The following describes three distinct motor mechanisms for *saccades*, *eye-carrying head motion*, and *gaze-stabilized head rotation*. For planning a gaze-shift, the brain has the luxury of choosing an arbitrary combination of these three mechanisms by determining the amount of their contribution and the pattern of their temporal implementation. The subject is initially fixating an arbitrary target and initial orientations of the eye and head are known variables of our problem:

(13) $${\vec{e}}_{i}\;=\;E_{i}\times {\vec{e}}_{r}$$

(14) $${\vec{h}}_{i}=H_{i}\;\times \;{\vec{h}}_{r}$$

(15) $${\vec{g}}_{i}=H_{i}\times E_{i}\;\times \;{\vec{g}}_{r}$$

#### Saccade

Saccade is the movement of the eyes relative to the head. For this movement the eye rotates in the head by rotation matrix ***Re*** and head stays fixed:

(16) $$\vec{e}=Re\;\times \;E_{i}\;\times \;{\vec{e}}_{r}$$

(17) $$\vec{h}=H_{i}\;\times \;{\vec{h}}_{r}$$

(18) $$\vec{g}=H_{i}\;\times \;Re\;\times \;E_{i}\;\times \;{\vec{g}}_{r}$$

#### Eye-carrying head rotation

Head is driven toward the target while no motor command is sent to eye muscles. Head rotates, moving eye with itself such that eye-in-head position remains unchanged (Guitton et al., 1984; Lehnen et al., 2008). Head and eye rotate together by unknown rotation matrix ***Rh***:

(19) $$\vec{e}=E_{i}\;\times \;{\vec{e}}_{r}$$

(20) $$\vec{h}=Rh\;\times \;H_{i}\;\times \;{\vec{h}}_{r}$$

(21) $$\vec{g}=Rh\;\times \;H_{i}\;\times \;E_{i}\;\times \;{\vec{g}}_{r}$$

#### Gaze-stabilized head rotation

This is the arbitrary movement of the head while gaze remains fixed in space (Lehnen et al., 2009a,b). Here, we assume the VOR causes eye-in-head motion to stabilize gaze. This type of eye movement is called vestibulo-ocular reflex. While head is rotating by unknown rotation matrix ***Rw***, eye is moving in the opposite direction by rotation matrix ***Rv***:

(22) $${\vec{e}}_{d}=Rv\;\times \;E_{i}\;\times \;{\vec{e}}_{r}$$

(23) $${\vec{h}}_{d}=Rw\;\times \;H_{i}\;\times \;{\vec{h}}_{r}$$

(24) $${\vec{g}}_{d}=Rw\;\times \;H_{i}\;\times \;Rv\;\times \;E_{i}\;\times \;{\vec{g}}_{r}$$

### Static kinematic model

**Figure 3:**
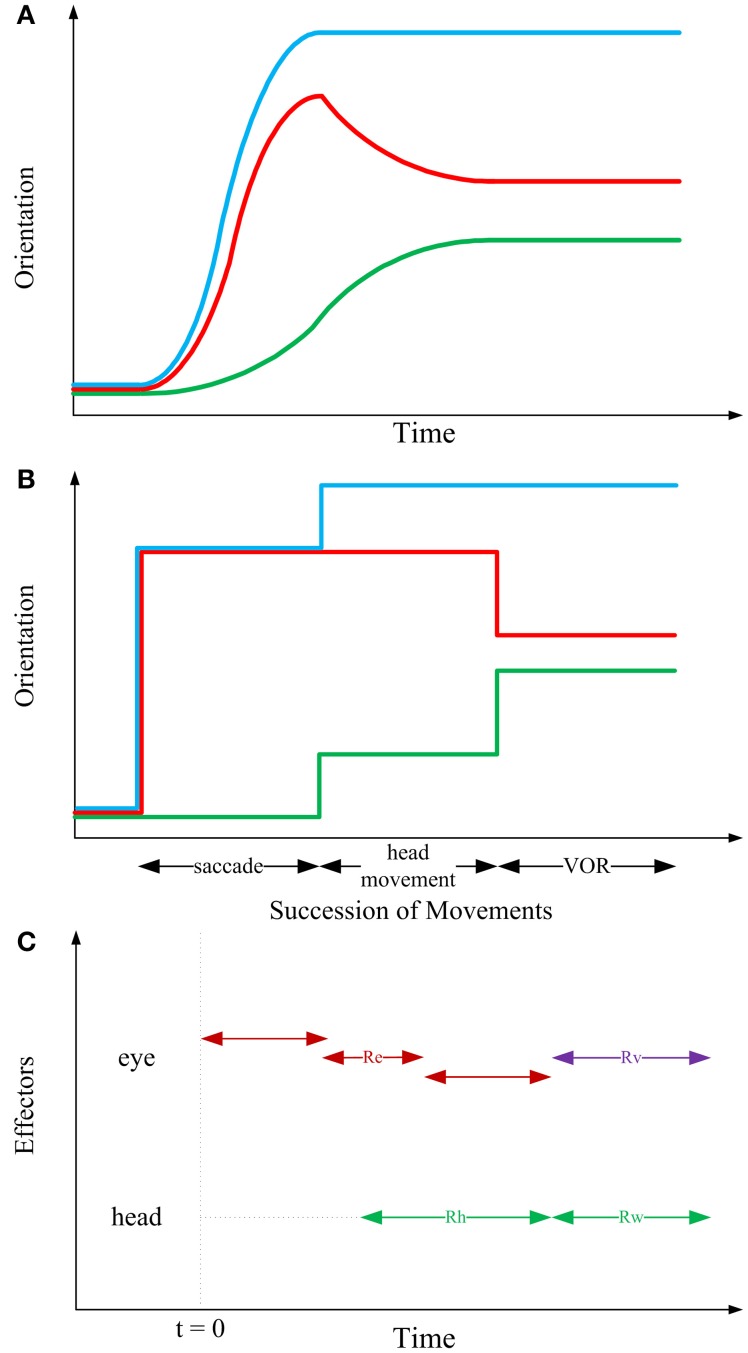
**Sequential structure of rotations in the kinematic model**. In the first two panels, blue, red, and green curves, respectively depict gaze, eye-in-head and head trajectories. **(A)** Typical 1-D behavioral diagram from the experiments on natural head-unrestrained gaze-shift (Guitton et al., 1990; Freedman and Sparks, 1997). This observed pattern has inspired the sequence of events devised in the static kinematic model. **(B)** Succession of movements in the kinematic model. Head remains fixed while the eye is moving in the head. Then, head rotates, moving eye with itself such that eye-in-head position remains unchanged; this rotation foveates the target. Then, head rotates to its definite position, while eye rotates in head to compensate for head movement and keep the target foveated. **(C)** Having solved the equations of the model based on our physiologically inspired assumptions and constraints, we find that the saccadic eye movement has its independent axis and can be implemented in any duration of time which ends before onset of VOR (red double-headed arrows). Onset of head movement is arbitrary but its two parts are implemented continuously after each other (green double-headed arrows). Eye rotation during VOR is implemented right at the time when the second part of head movement is happening (violet double-headed arrow).

As it is experimentally observed and schematically illustrated in Figure 3A, the gaze-shift typically begins when the saccadic eye movement rapidly changes the positions of the eyes relative to the head and it ends when the line of sight is directed toward the visual target. The rapid eye movement component of the gaze-shift ends at approximately the same time. The head continues moving toward the target while the eyes move in the opposite direction at a velocity that is approximately the same as that of the head. As a result, the direction of the line of sight remains stable (Bizzi et al., 1971, 1972; Zangemeister et al., 1981).

According to observations of visual orienting behavior it is clear that movements of the eyes and head can begin at approximately the same time. However, recording the activity of neck and eye muscles reveals that even when movement onsets are synchronous, the command to move the head precedes the command to move the eyes (Bizzi et al., 1971; Zangemeister et al., 1981; Corneil et al., 2007). Furthermore, inspection of the behavior over a broad range of gaze-shift amplitudes, task requirements, and target predictability indicates that the relative timing of eye and head movements is variable depending on task and training (Zangemeister and Stark, 1982; Guitton and Volle, 1987; Freedman and Sparks, 1997; Crawford et al., 1999): the head can lag the onset of eye movements during small amplitude gaze-shifts, but during large amplitude movements, or movements to target locations that are predictable head movements can begin well-before saccades. Electrical stimulation in the omnipause neuron region can delay saccade onset without altering the initiation of head movements (Gandhi and Sparks, 2007); evidence that the triggering mechanisms for the eyes and head are not shared.

According to the evidence for temporal coupling of eye and head movements described above, a separation (at least with respect to movement initiation) of head and eye command signals can be identified within the brainstem structures that control coordinated eye–head movements. This may indicate that the brain plans a gaze-shift at different levels, i.e., kinematics vs. dynamics. Accordingly, inspired by the fundamentals of engineering design, we propose that a complete model of gaze-shift is planned in three levels of information processing. At the highest level, the static kinematic model illustrated in Figure 3B, we define a set of movements as a framework for computing the early motor commands for eye and head. At the middle level, sketched in Figure 3C, a temporal structure for implementation of these movement commands should be proposed. It can be shown that these two levels are independent, i.e., succession used for computation of motor commands does not dictate the timing of their implementation. Rather, saccadic eye movement can start before or after the onset of head movement and can finish well-before the onset of VOR (Figure 3C). At the lowest level, the required torques are calculated by putting the then-known kinematic variables in governing conservation equations, and then, knowing the structure of motoneurons and muscles, the required neural signals could be derived. Having emphasized this hierarchical structure, in this paper, we are only concerned with the higher level.

As it is shown in Figure 3B, our proposed higher-level kinematic strategy consists of three stages and systematically combines the three previously mentioned motor mechanisms. In the first stage, head remains fixed while the eye is moving in the head. In the second stage, head rotates, moving eye with itself such that eye-in-head position remains unchanged; this rotation foveates the target. In the third stage, head rotates to its definite position, while the eye rotates in the head to compensate for head movement and keep the target foveated (VOR). Table 1 shows the orientations of the eye, head and gaze after any of the three stages of the model.

**Table 1:** **Mathematical description of eye, head, and gaze orientations at different stages of model**.

	***$\vec{e}$***	***$\vec{h}$***	***$\vec{g}$***
Initial condition	***E***_***i***_ × $\vec{e}$_*r*_	***H***_***i***_ × $\vec{h}$_*r*_	***H***_***i***_ × ***E***_***i***_ × $\vec{g}$_*r*_
After first stage	***Re*** × ***E***_***i***_ × $\vec{e}$_*r*_	***H***_***i***_ × $\vec{h}$_*r*_	***H***_***i***_ × ***Re*** × ***E***_***i***_ × $\vec{g}$_*r*_
After second stage	***Re*** × ***E***_***i***_ × $\vec{e}$_*r*_	***Rh*** × ***H***_***i***_ × $\vec{h}$_*r*_	***Rh*** × ***H***_***i***_ × ***Re*** × ***E***_***i***_ × $\vec{g}$_*r*_
Desired condition	***Rv*** × ***Re*** × ***E***_***i***_ × $\vec{e}$_*r*_	***Rw*** × ***Rh*** × ***H***_***i***_ × $\vec{h}$_*r*_	***Rw*** × ***Rh*** × ***H***_***i***_ × ***Rv*** × Re × *E*_***i***_ × $\vec{g}$_*r*_

Thus, desired orientations can be written as a function of initial orientations and the rotations:

(25) $$E_{d}=Rv\times Re\times E_{i}$$

(26) $$H_{d}=Rw\times Rh\times H_{i}$$

(27) $$G_{d}=Rw\times Rh\times H_{i}\times Rv\times Re\times E_{i}$$

### Solving the static model

#### Dependence of desired head position on desired gaze position

When the desired target appears in the visual field, the main signal for planning the gaze-shift and the main known input of our model is constructed in the form of the 2-D desired angular gaze position, $\vec{c}$_*d*_. We define the parameters α, β to determine how much the head would move, in horizontal and vertical directions, respectively, relative to initial head position. Setting α, β to zero, the model is reduced to a model of head-fixed gaze-shift. Model parameters α, β determine how the 2-D desired angular head position $\vec{b}$_*d*_ would be derived from $\vec{c}$_*d*_ and the initial conditions:

(28) $${\vec{b}}_{d}=\left[\begin{array}{c} b_{i}^{Y}+\alpha \times \left(c_{d}^{Y}-b_{i}^{Y}\right)\\ b_{i}^{Z}+\beta \times \left(c_{d}^{Z}-b_{i}^{Z}\right) \end{array}\right]$$

Where 0 < α <1 and 0 < β <1. Desired gaze and head vectors, $\vec{g}$_*d*_ and $\vec{h}$_*d*_, can then be derived from $\vec{c}$_*d*_ and $\vec{b}$_*d*_ based on Equations (7) and (8).

#### Fick constraint for head orientation

Fick system represents a general rotation as successive rotations with magnitudes θ, φ, ψ about local vertical, horizontal and torsional axes, respectively. Rotation matrix in Fick system is:

(29) $$\left[\begin{array}{ccc} \text{cos}\left(\theta \right)\text{cos}(\varphi ) & \text{cos}\left(\theta \right)\sin\left(\psi \right)-\text{sin}\left(\theta \right)\text{cos}\left(\psi \right) & \cos\left(\theta \right)\sin\left(\varphi \right)\text{cos}\left(\psi \right)\text{cos}\left(\psi \right)\text{+sin}\left(\theta \right)\sin(\psi )\\ \sin\left(\theta \right)\text{cos}(\varphi ) & \sin\left(\theta \right)\sin\left(\varphi \right)\sin\left(\psi \right)\text{+ cos}\left(\theta \right)\text{cos}\left(\psi \right) & \sin\left(\theta \right)\sin\left(\varphi \right)\text{cos}\left(\psi \right)-\text{cos}\left(\theta \right)\text{sin}(\psi )\\ -\text{sin}(\varphi ) & \text{cos}(\varphi )sin\left(\psi \right) & \text{cos}(\varphi )cos(\psi ) \end{array}\right]$$

It has been shown that after a natural head-free gaze-shift, desired head orientation obeys the Fick constraint. This constraint states that if one represents **H_d_** in the Fick system, then the torsional component of this representation is zero:

(30) $$H_{d}=\;\left[\begin{array}{ccc} \text{cos}\left(\theta \right)\text{cos}(\varphi ) & -\sin\left(\theta \right) & \cos\left(\theta \right)\sin\left(\varphi \right)\\ \sin\left(\theta \right)\text{cos}(\varphi ) & \text{cos}\left(\theta \right) & \sin\left(\theta \right)\sin\left(\varphi \right)\\ -\sin(\varphi ) & 0 & \text{cos}(\varphi ) \end{array}\right]$$

Knowing $\vec{h}$_d_, Fick angles of desired head orientation, θ, φ, can be derived based on general relation (Equation 2) and the Equation (30):

(31) $$\varphi =\;\sin^{-1}(-h_{d}^{Z})$$

(32) $$\theta =\sin^{-1}\left(\frac{h_{d}^{Y}}{\cos(\sin^{-1}(h_{d}^{Z}))}\right)$$

So, desired head orientation ***H***_***d***_ would now become known to us.

#### Uniqueness of head rotation command

From observations of the behavior in head-unrestrained experiments, it has been seen that only one head rotation command is implemented during one planned gaze-shift. However, two distinct measures of the head movement have been defined: the total movement of the head from start to finish and the amount that the head movement contributed to the accomplishment of the gaze-shift, often referred to as the head contribution (Bizzi et al., 1972; Morasso et al., 1973). So, in our model structure, we assume that the head rotations in the first and second stages of our model are just two successive parts of one head rotation ***Rt***:

(33) Rt=Rw×Rh

This means that ***Rw*** and ***Rt*** have the same axis of rotation:

(34) $${\vec{u}}_{Rt}={\vec{u}}_{Rw}={\vec{u}}_{Rh}$$

Where *u* is the axis of rotation and rotation magnitudes of ***Rh*** and ***Rw*** are complementary fractions of τ_***Rt***_:

(35) $$\tau_{Rh}=\;\delta \times \tau_{Rt}$$

(36) $$\tau_{Rw}=\;(1-\delta )\times \tau_{Rt}$$

Where τ is the magnitude of rotation, 0 < δ <1, and δ is a model parameter which could depend on different factors, most importantly the total head rotation. After finding ***H***_***d***_ from Equations (30–32), we can derive **R*t*** based on Equations (26) and (33):

(37) $$Rt=H_{d}\times H_{i}{}^{-1}$$

***Rh*** and ***Rw*** will be found as we know their axis and magnitude of rotation.

#### Listing's law for eye orientation

***H***_***d***_ and $\vec{g}$_*d*_ being known, we can find $\vec{e}$_*d*_ from:

(38) $${\vec{e}}_{d}=H_{d}{}^{-1}\times {\vec{g}}_{d}$$

Based on Listing's law, if one represents eye-in-head orientation by the classical magnitude/axis convention, then the axis of rotation would always be in the Listings plane (LP). LP is a plane fixed to the head and rotating with it. LP is orthogonal to the straight ahead sight/gaze axis. According to this constraint, the third component of the unit vector, which denotes the axis of rotation for eye-in-head orientation matrix, is zero. For the desired eye-in-head orientation:

(39) $${\vec{u}}_{E_{d}}=[0;;u_{E_{d}}^{y};;u_{E_{d}}^{z}]$$

(40) $$\text{​​​​​​​​​​​​​​​​​​​​​​​​​​}E_{d}=\left[\begin{array}{ccc} cos\left(\tau_{E_{d}}\right) & -u_{E_{d}}^{z}\times sin(\tau_{E_{d}}) & u_{E_{d}}^{y}\times sin(\tau_{E_{d}})\\ u_{E_{d}}^{z}\times sin(\tau_{E_{d}}) & cos\left(\tau_{E_{d}}\right)+u_{E_{d}}^{y}{}^{2}\times (1-cos\left(\tau_{E_{d}}\right)) & u_{E_{d}}^{y}\times u_{E_{d}}^{z}\;\times \left(\text{1}-cos\left(\tau_{E_{d}}\right)\right)\\ -u_{E_{d}}^{y}\times sin(\tau_{E_{d}}) & u_{E_{d}}^{y}\times u_{E_{d}}^{z}\;\times \left(\text{1}-cos\left(\tau_{E_{d}}\right)\right) & cos\left(\tau_{E_{d}}\right)+u_{E_{d}}^{z}{}^{2}\times (1-cos\left(\tau_{E_{d}}\right)) \end{array}\right]$$

Substituting Equation (40) into Equation (1) and knowing $\vec{e}$_*d*_ from Equation (38), we can solve the system of equations for *u*^*y*^_***E***__***d***_ and *u*^*z*^_***E***__***d***_ and τ_*E*_*d*__:

(41) $$\tau_{E_{d}}=\;\cos^{-1}\left(e_{d}^{x}\right)$$

(42) $$u_{E_{d}}^{y}=\;-e_{d}^{z}/sin(\tau_{E_{d}})$$

(43) $$u_{E_{d}}^{z}=\;e_{d}^{y}/\sin(\tau_{E_{d}})$$

So, from Equation (40), we have ***E***_***d***_. Let's define rotation matrix ***Ra*** as:

(44) Ra=RvRe

Knowing ***E***_***i***_ and ***E***_***d***_, we can derive ***Ra*** from Equation (25):

(45) $$Ra=E_{d}\times E_{i}{}^{-1}$$

#### Gaze stability during VOR

We are assuming that the retinal image is perfectly stabilized during the third stage of the model and by the execution of ***Rw*** and ***Rv***. Then, by looking at Table 1, we have:

(46) $$Rh\times H_{i}\times Re\times E_{i}=Rw\times Rh\times H_{i}\times Rv\times Re\times E_{i}$$

From Equation (46), we can derive ***Rv*:**

(47) $$Rv=H_{i}{}^{-1}\times Rh^{-1}\times Rw^{-1}\times Rh\times H_{i}$$

Knowing ***Ra*** and ***Rv***, ***Re*** can be derived from Equation (44):

(48) $$Re=Rv^{-1}\times Ra$$

Therefore, all the unknown parameters of the model have been derived from the governing equations of the model considering the assumptions and constraints.

#### Simulation of full movement trajectories

The model described above was designed to simulate the key kinematic events in the gaze shift illustrated in Figure 3B. For simulation purposes, this was sufficient to show initial and final eye (saccade and VOR) and head movement positions. A complete dynamic model of the system would require neural and mechanical elements downstream from the model in Figure 1, and goes beyond the goals and scope of the current paper. However, for some of the simulations shown below it was desirable esthetically or scientifically to show intermediate points along the entire trajectory. In brief, to do this we assumed constancy of the axis of rotation for all eye and head motions except VOR (whose axis of rotation is determined online from the online spatial orientation of head). We then discretized the magnitude of rotation based on specific growth functions in a time-frame illustrated in Figure 3C. The 3-D constraints in our model were applied on initial and final eye/head orientation and we do not analyze velocity or acceleration in this paper, so, the details of these growth functions have no bearing on any of the questions asked here.

## Results and discussion

Here we test the model by comparing its simulated output to previously reported or expected performance of the real system in several different tasks. Unless otherwise stated, the model parameters are set to α = β = δ = 0.5, i.e., midway in their possible range of 0–1.

### Gaze accuracy and the 3-D reference frame transformations

It has been shown both with saccade simulations (Crawford and Guitton, 1997b) and real saccade data (Klier and Crawford, 1998) that retinal error only corresponds directly to the gaze movement vector for saccades directed toward, across, or away from Listing's primary eye position. For all other saccades, retinal error needs to be mapped onto different saccade vectors as a function of initial eye orientation. This is simply a function of the geometry of the system; it cannot be any other way. However, failure to properly account for this, in our model (or the real gaze control system), would result in saccade errors that increase with the position component and length of retinal error (which did not occur). This has not been measured behaviorally with head-unrestrained gaze shifts, but the predicted errors here would be so large (up to 90°) that the errors would be obvious in daily life if the brain did not account for this. Moreover, the converse has been shown with simulations and experiments: a single retinal vector evoked from stimulation of the brain (e.g., in superior colliculus) results in very different eye-head gaze trajectories as a function of initial eye orientation (Klier et al., 2001; Martinez-Trujillo et al., 2004).

**Figure 4:**
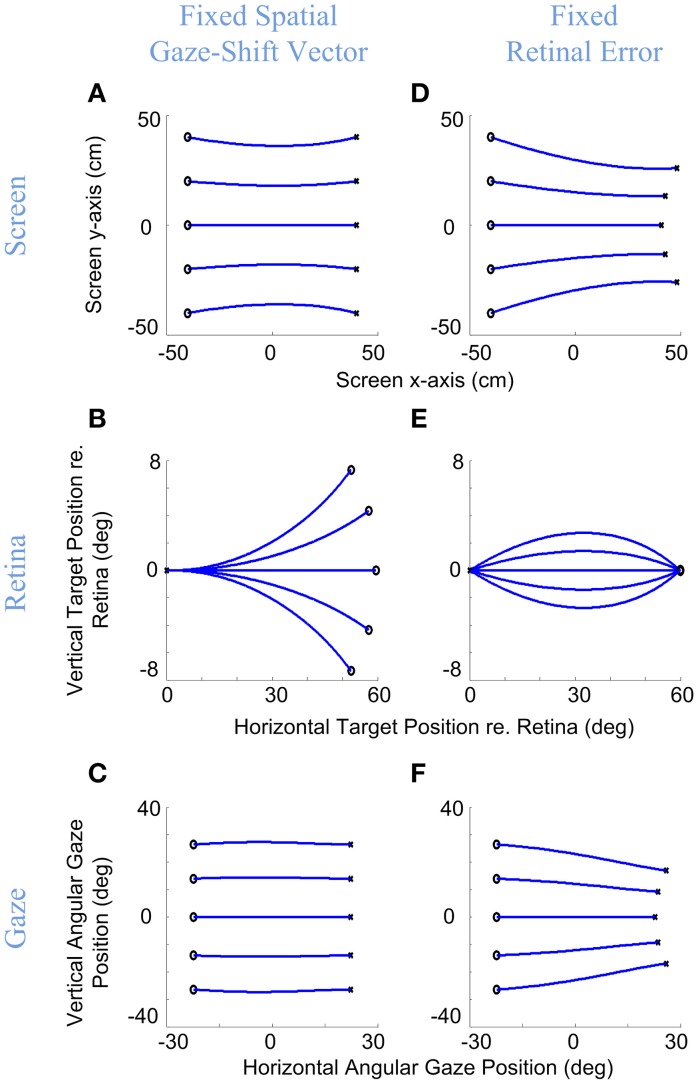
**Gaze accuracy and the 3-D reference frame transformations for gaze-shifts**. Rightward gaze-shifts are simulated from five different vertical altitudes with either a fixed symmetric horizontal gaze-shift, −40 cm left to 40 cm right, on a flat target screen **(A–C)**, or from the same initial positions with a fixed retinal error of 60° right **(D–F)**. First row shows the initial and desired target positions on the screen and the development of gaze direction on the screen during the gaze-shift. Second row shows the development of the target position in retinal coordinates during gaze-shift. Third row shows the development of the 2-D angular gaze position during gaze-shift. For both conditions, the model parameters are set to α = β = δ = 0.5. Circles show initial target locations while stars show the desired positions of target. Note that in **(B)** even though the targets are due right in spatial coordinates, they have variable vertical components in retinal coordinates, whereas conversely retinal errors in **(E)** start and end at the same positions, and correspond to different gaze trajectories.

We have simulated this behavior with our model in Figure 4. Here, the model generated rightward gaze-shifts from different vertical positions but the same horizontal components (°), rightward gaze trajectory toward the symmetric target on the opposite side (left column) or with a fixed rightward retinal error input (right column). The intersection point of gaze on a forward-facing target screen is shown in the top row (with end points shown as ×), the instantaneous points of stimulation of the corresponding positions on the retina (initial retinal error being the main model input) are shown in the middle row, and the resulting angular gaze trajectories (the model output) are shown in the bottom row. The trajectories in the upper and bottom rows are very similar, starting from the left and proceeding right. The trajectories in the middle row proceed in the opposite direction because the desired targets start to the right in retinal coordinates (dispensing with the optical inversion) and then proceed to the left as they converge toward (0, 0), i.e., the retinal coordinates of the fovea. This indicates the accuracy of the model in bringing the image of the desired target to the fovea.

More importantly, these simulations illustrate the non-trival relationship between retinal error vectors and gaze shift direction, and the ability of our model to handle this. As the left column shows, when the target is due right of initial gaze position (Figure 4A), this corresponds to non-horizontal retinal errors (Figure 4B) as a non-linear function of initial vertical position, but the model correctly converts this into rightward gaze shifts (Figure 4C). Conversely, the right column shows that a rightward retinal error (Figure 4E) corresponds to different directions of target position relative to initial position (Figure 4D), but again the correct movement trajectory is generated (Figure 4F). We obtained analogous results for every combination of retinal error and position that we tested. There can be no linear trivial mapping between the retina and motor output, unless one models the pulling actions of the eye and neck muscles into retinal coordinates and aligns the centers of rotation of the eyes and head, which is not realistic. Thus, the model must (and does) perform an internal reference frame transformation, based on its retinal inputs and its eye/head orientation inputs.

### Eye, head, and gaze orientations and their constraints

Donders' law, as originally stated, suggested that the eye should only attain one torsional orientation for each gaze direction, irrespective of the path taken to acquire that position. This rule has since been applied and elaborated to a number of situations and more specific rules. Behavioral data from 3-D head-fixed and head-free tasks (Glenn and Vilis, 1992; Radeau, 1994; Crawford et al., 1999) have shown that (1) orientation of eye relative to head at the end of the gaze-shift lies in the Listing's plane and has zero torsional component, (2) the final orientation of head relative to shoulder obeys the Fick law, i.e., the torsional component of head orientation in Fick system is zero, and (3) the orientation of the eye-in-space during gaze fixations also adheres to a form of Donder's law similar to the Fick rule.

**Figure 5:**
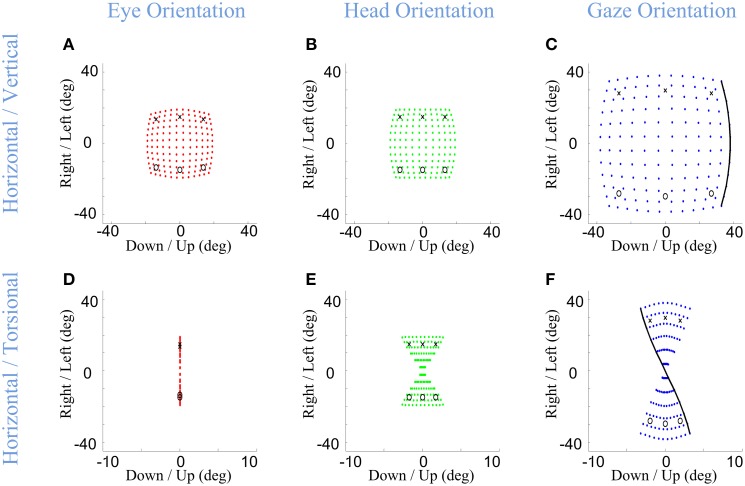
**Distributions of head, eye, and gaze orientations for equal contributions of eye and head rotations to horizontal and vertical directions**. Model simulations producing gaze-shifts from the central fixation point (reference condition) to a uniform distribution of targets on the screen in range (−40, 40) degrees horizontal and (−40, 40) degrees vertical. The first **(A,D)**, second **(B,E)**, and third **(C,F)** columns, respectively, show eye-in-head (red), head-in-space (green), and eye-in-space orientations after the gaze-shift. First row illustrates the horizontal (right/left) against the vertical (up/down) components while the third row shows the horizontal (right-left) against the torsional (CW/CCW) components. The parameters of the model are set to α = β = δ = 0.5. The black curve shows gaze orientations for targets aligned horizontally on top of the screen.

Importantly, in our model, the Listings and Fick constraints on final eye and head orientation were directly implemented, whereas the torsion of the eye in space was an emergent property of the above constraints. What would this look like? The final positions of gaze-shifts of various amplitudes and directions are simulated in Figure 5 for the eye-in-head (left column), head-in-space (middle column), and eye-in-space (right column), where the first row shows the 2-D components of this range and the second row shows horizontal position plotted as a function of torsional position. As one can see in Figure 5D, irrespective of the magnitude or direction of eye or head rotations during gaze-shifts, this kinematic model always produces a final eye-in-head orientation that obeys Listing's law, forming a flat range of zero torsional positions. In contrast, Fick constraint manifests as a bow-tie shape of the distribution of head orientations in horizontal-torsional rotation plane. As one can see in Figure 5E, all final head positions, irrespective of the magnitude or direction of head rotation, obey the Fick law for head orientation. A similar, but less pronounced, Fick-like twist in the range of final orientation is seen for the eye in space (Figure 5F). In other words, in our model the Fick range of eye orientation in space was an emergent property of the eye and head constraints implemented in our model.

### Development of the eye, head, and gaze orientations during gaze-shift

The previous section only described end point kinematics of the entire eye-head gaze shift. The 3-D trajectories of the eye and head during motion, and their relationship to the end-point constraints, are potentially much more complex. It is generally agreed that Listing's law is obeyed during head-restrained saccades (Ferman et al., 1987b; Tweed and Vilis, 1990), although small torsional “blips” near the ends of the trajectories have been scrutinized to test the role of eye mechanics in implementing the position-dependent “half angle rule” that describes 3-D eye velocities for Listing's law (Straumann et al., 1995, 1996). We have assumed that these rules are perfectly implemented downstream from the output of our model so our model cannot predict any such “blips.” However, eye trajectories become much more complicated in the head-unrestrained situation because saccades must be coordinated with the VOR, which does not obey Listing's law, resulting in large transient deviations of eye position from Listing's plane (Crawford and Vilis, 1991; Tweed et al., 1998; Crawford et al., 1999; Klier et al., 2003). Likewise, during rapid gaze shifts in monkeys the head appears to deviate from the static Fick constraint when it takes the shortest path between two points on the curved Fick range (Crawford et al., 1999). These saccade/VOR behaviors have been considered in a previous modeling study (Tweed, 1997), but not the above-mentioned head behavior.

**Figure 6:**
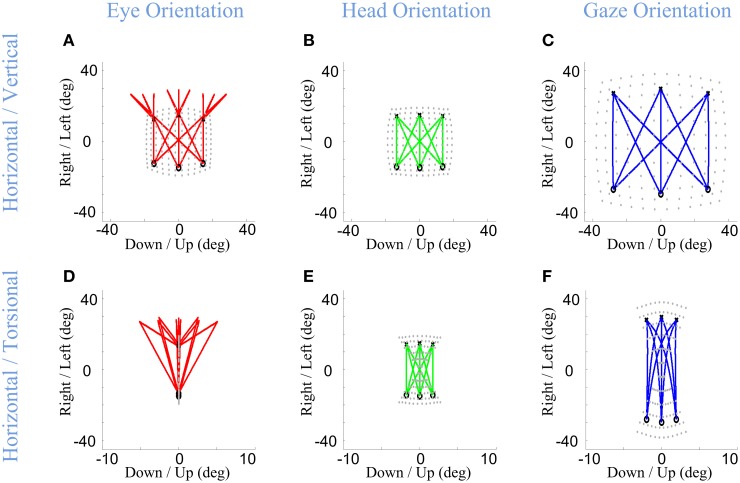
**Spatial path of the development of eye, head, and gaze orientations during gaze-shift**. Three example gaze-shifts have been planned from three targets, vertically aligned at −40 cm on the screen, to another three targets, vertically aligned at 40 cm. The locations of eye, head, and gaze in initial condition are shown by circles while their locations in desired condition are shown by crosses. First **(A–C)** and second **(D–F)** rows show the temporal development of eye, head, and gaze in vertical-horizontal and torsional-horizontal planes, respectively.

**Figure 7:**
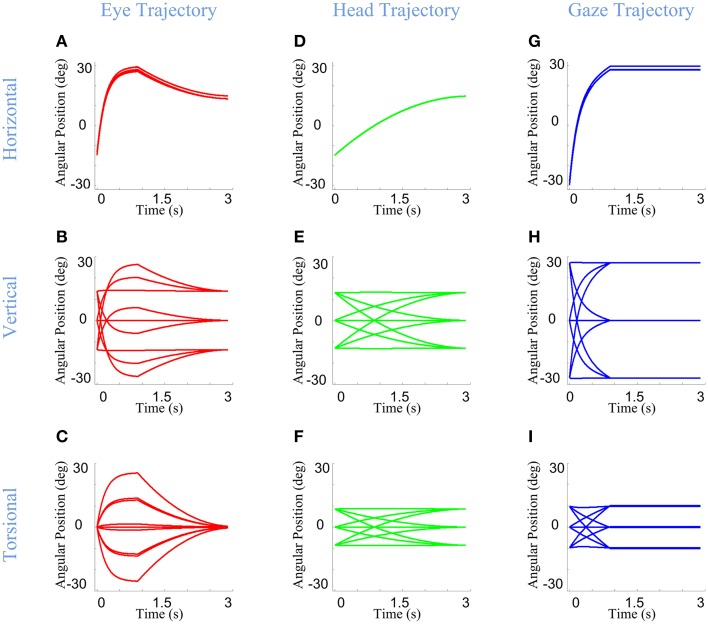
**Temporal pattern of development of eye, head, and gaze orientations during gaze-shift**. For the same nine gaze-shifts, between two groups of vertically aligned targets, we have shown the development of the orientations. First **(A–C)**, second **(D–F)**, and third **(G–I)** columns show orientations of eye, head, and gaze, respectively. First, second, and third rows describe the development of horizontal, vertical, and torsional components of orientations, respectively.

Here, we consider the ability of our model to simulate these behaviors, based on its static implementation of the Listing and Fick rules, and the simple temporal discretization of trajectories described in Section Model Formulation. Figure 6 shows example eye, head, and gaze trajectories between three initial (°) and final (×) gaze positions (corresponding to the same symbols/positions shown in Figure 5). Figure 6 thus shows the development of gaze-shift, in different rotation planes, between two groups of vertically aligned targets on the screen. Likewise, Figure 7 illustrates the temporal development of the horizontal (upper row), vertical (middle row) and torsional (bottom row) components of eye (left column), head (middle column) and gaze (right column) orientations during the same set of gaze shifts as shown in Figure 6.

First, we consider the eye-in-head behavior. In real time, the VOR is evoked through vestibular stimulation after the saccade, but in our model (and we propose in real physiology) the brain implicitly predicts the VOR from intended head movement signals in order to program the right amount of torsion and also brings the eye onto the correct final 2-D orientation (Crawford and Guitton, 1997a; Misslisch et al., 1998). This is illustrated in the left columns of Figures 6, 7. Eye orientation relative to head goes out of its range during saccade and comes back to the planned configuration by VOR (Figure 6A). Particularly, the eye-in-head torsion starts at the Listing's plane, deviates from the LP during the saccade and gets back into the LP by the VOR (Figure 6D). The reasons for this are more clearly illustrated in Figure 7. Here, one can see that the gaze-shift is implemented in two time phases: (1) Eye undergoes a saccade, head contributes to gaze, and gaze is placed on the target. (2) Head undergoes its second-stage movement (canceled out by the VOR), the eye is driven by the VOR, and gaze is stabilized. The eye torsion (Figure 7C) starts at zero which indicates that initial eye orientation obeys the Listing's law. Thus, torsions in these two phases neutralize each other such that the torsion of the final eye orientation is zero in Listing's plane coordinates. Similar principles hold for horizontal and vertical eye position (Figures 7A,B), except that these saccade components are larger than the corresponding VOR components. This replicates the behaviors observed in monkey and human gaze shifts (Crawford and Vilis, 1991; Tweed et al., 1998; Crawford et al., 1999; Klier et al., 2003).

In our model, the head's Fick constraint is only explicitly specified at its initial and final positions, and the head is moved uniformly through the gaze shift by a single rotation command. As a result, in our simulations, the head starts and ends in the Fick range, moves smoothly between these positions, and often violates the Fick constraint during the movement (Figures 6E, 7F). The deviations from Fick are made clear by comparing Figures 5E, 6E, which has been imposed in gray beneath Figure 6E for easy reference. If the head always obeyed the Fick constraint during gaze-shifts, it would take a path passing through the bow-tie shape. Instead, the head takes an almost direct path between the two Fick-obeying points. This is most clear in the head movements between corners with similar torsion (e.g., the two left-side corners and two right side corners in Figure 6E), where the head completely leaves the normal Fick range. This replicates the experimental observations in the monkey (Crawford et al., 1999). However, more experiments are required to know if the head always follows the same strategy.

Finally, note again that in our model, gaze (eye orientation in space) torsion is also not explicitly controlled during the trajectory, but is rather an emergent property (roughly the simultaneous sum) of eye and head torsion during the gaze shift. Thus, not surprisingly, gaze torsion also deviates from its normal qausi-Fick range during the gaze shift (Figures 6F, 7I).

### Eye-head coordination strategies influence eye-in-space orientation

During visual fixations, the entire 3-D range of eye orientation is important because this determines the orientation of the retina relative to the visual world (Ronsse et al., 2007). However, this topic (eye orientation in space) has received surprisingly little attention compared to 2-D gaze direction. Our physiologically-inspired model assumes that eye-in-space torsion is an emergent property of separate constraints on eye and head torsion. As we shall see, this gives rise to the possibility that eye-head coordination strategies could interact with these constraints to produce different ranges of eye orientation in space. In this section we consider several possible, experimentally testable situations where this could occur.

It has been shown in many experiments (and is also intuitively obvious from personal experience) that the amount that the head rotates for a constant gaze-shift changes depending on many factors, including initial head orientation (Guitton and Volle, 1987), visual range (Crawford and Guitton, 1997a), behavioral context (Land, 1992; Khan et al., 2009), expected future gaze targets (Monteon et al., 2012), and inter-subject differences. In order to reflect this variability, we varied α & β (which respectively, respectively, determine the horizontal and vertical angular positions on which head falls after the gaze-shift) along the range (0, 1). This allowed us to explore the kinematic consequences of (1) utilizing different overall eye vs. head contributions to gaze-shift, and (2) differential vertical vs. horizontal contributes of the head to gaze-shift.

**Figure 8:**
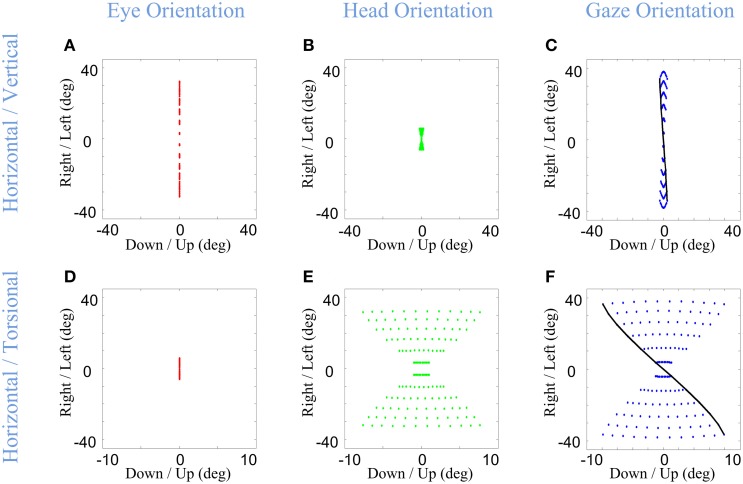
**Distributions of head, eye, and gaze orientations for two extreme cases of almost only eye contribution (head-fixed Saccade) and almost only head contribution**. We have made the model to plan gaze-shifts from the central fixation point (reference condition) to a uniform distribution of targets on the screen in range (−40, 40) degrees horizontal and (−40, 40) degrees vertical. Eye-in-head (first column in red), head-in-space (second column in green), and eye-in-space (third column in blue) orientations are illustrated. Only the horizontal (right-left) against the torsional (CW/CCW) diagrams are included in this figure. The parameters of the model for the first row **(A–C)** is set to α = β = 0.15 and δ = 0.5 while for the second row **(D–F)** they are set to be α = β = 0.85 and δ = 0.5. The black curve shows gaze orientations for targets aligned horizontally on top of the screen.

Infinitesimal values of α & β correspond to nearly head-fixed saccades saccades (Figures 8A–C), reflecting situations such as watching television and reading (Proudlock et al., 2003). Here, eye orientation occupies almost the same area as gaze (Figure 8A vs. Figure 8C) while head orientation is limited to a very small area (Figure 8B). In this condition, gaze orientation comes very close to Listing's law (Figure 8C). In contrast, large values of α & β (Figures 8D–F) were used to simulate the situation where final head orientations occupied almost the same area as gaze distribution, and eye-in-head orientation returns to a returns to a central range near primary position after the VOR. This emulates behavioral situations such as driving a car (Land, 1992) and certain experiments in which subjects were required to rotate their head more (Ceylan et al., 2000). Here, the head's greater contribution to gaze orientation (while maintaining final eye-in head torsion at zero) results in a Fick-like range of eye-in-space orientations identical to that of the head (Figure 5F), and thus more “twisted” than observed when the eye and head contribute equally.

Note that the latter simulations assumed that constraints on eye and head orientation are not influenced by these different eye head coordination strategies. To our knowledge, this has not been directly tested for the “eye-only” situation, but, experimental studies that increased the amount of head orientation to equal gaze orientation (by training subjects to look through a head-fixed “pinhole” or point a head-fixed light toward the target) caused the head to develop a more Listing-like strategy (Crawford et al., 1999; Ceylan et al., 2000) and thus producing a less twisted eye-in-space range. This could be simulated here by replacing our head's Fick constraint with a Listing's law constraint as used in the eye pathway. The more important point is that the Ceylan et al. (2000) study concluded that these head constraints are purely motor, whereas the current analysis suggests that their result might have been related to orientation of the eye in space and its implications for vision. If so, then the brain would have to be aware of the interactions between eye-head coordination and 3-D orientation constraints, and alter the latter accordingly to achieve the right position range.

**Figure 9:**
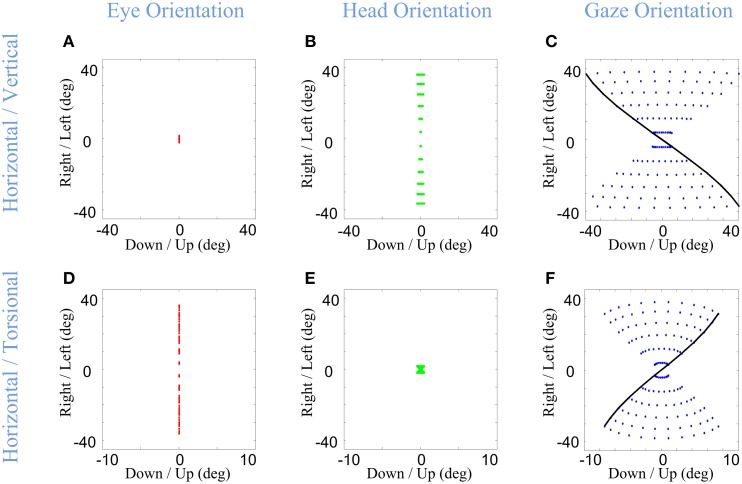
**Distributions of head, eye, and gaze orientations for two extreme cases of almost only head contribution to horizontal gaze-shift or almost only head contribution to vertical gaze-shift**. We have made the model to plan gaze-shifts from the central fixation point (reference condition) to a uniform distribution of targets on the screen in range (−40, 40) degrees horizontal and (−40, 40) degrees vertical. Eye-in-head (first column in red), head-in-space (second column in green), and eye-in-space (third column in blue) orientations are illustrated. The horizontal (right-left) against the torsional (CW/CCW) diagrams are only included in this figure. The parameters of the model for the first row **(A–C)** is set to α = 0.05, β = 0.95, and δ = 0.5 while for the second row **(D–F)** they are set to be α = 0.95, β = 0.05, and δ = 0.5. The black curve shows gaze orientations for targets aligned horizontally on top of the screen.

Another interaction between eye-head coordination and orientation constraints is perhaps more surprising, and yet inevitable if the assumptions behind our model are correct. It has been experimentally observed that the contribution of the head to the gaze-shift can be different in horizontal and vertical directions, usually providing more horizontal contribution (Freedman and Sparks, 1997; Crawford et al., 1999). Figure 9 shows the ability of the model to plan such distinct gaze-shifts, and uses these simulations to illustrate how relative vertical-horizontal contributions of the head to gaze shifts could have a profound influence on orientation of the eye in space.

In the first row of Figure 9, the eye (Figure 9A) contributes mainly to vertical component (not shown) and the head (Figure 9B) is mainly contributing to the horizontal component of the gaze-shift. This essentially reduces eye and head orientation each to rotation about two fixed axes and a one-dimensional range, but results in a strong “Fick-like” twist in the eye-in-space orientation range (Figure 9C), even stronger than in our default simulations (Figure 5F). This is because here we have essentially turned the system into a true Fick Gimbal, where the head rotates about a body-fixed vertical axis and the eye rotates about a head-fixed horizontal axis. This supports the notion that the relatively larger contribution of the head to horizontal rotation in most situations contributes to the Fick-like range of eye-in-space (Crawford et al., 1999).

In the second row of Figure 9, the directional contributions of the head and eye have been reversed: the eye mainly rotates horizontally about a vertical axis and the head mainly rotates vertically (not shown) about a horizontal axis. Physically, this now resembles a Helmholtz system, where the vertical axis is embedded on a fixed horizontal axis. This results in a range of eye-in-space orientations (Figure 9F) with an opposite twist to what we have seen so far, in other words, the opposite amount of torsion for a given gaze direction. This simulation predicts that if subjects can be induced to make gaze shifts with pure vertical head rotation, they should develop a similar range of eye-in-space orientation, unless constraints on torsion are modified in some way that has not yet been observed. This prediction could be easily tested by instructing a subject to use the head vertically or horizontally in a gaze shift. In the event that subjects do switch to the Helmholtz constraint, this would be strong support for our model.

Thus, even if one assumes that 2D eye-head coordination and 3D eye/head constraints are implemented independently (as we have assumed here), they still interact in complex ways to influence 3D eye-in-space torsion as a function of 2D gaze direction. Since all three components of eye orientation (horizontal, vertical, and torsional) interact with 2D visual stimulus direction in a complex non-linear fashion to determine the retinal location of visual stimulation (Crawford and Guitton, 1997b; Henriques and Crawford, 2000; Blohm and Crawford, 2007), this has non-trivial implications for vision. First, it has been shown previously that the brain accounts for 3D eye orientation in decoding patterns of visual stimulation in some behaviors (Henriques et al., 1998; Klier and Crawford, 1998; Blohm and Crawford, 2007; Blohm et al., 2008), but this has not been tested in the situations simulated here. Second, it is possible that patterns of eye-head coordination are chosen to simplify or optimize patterns of retinal stimulation. Third, it is known that (contrary to the simplifying assumptions above) 3-D torsional constraints on the head are sometimes altered for different patterns of 2-D eye-head coordination (Crawford et al., 1999; Ceylan et al., 2000). This suggests the possibility that implementation of 2-D eye-head coordination and 3-D constraints might be linked in some way as to optimize vision. In short, our simulations highlight a large potential for experimental studies of the relationships between eye-head coordination and vision.

## Concluding remarks

We have proposed a kinematic model that plans accurate and coordinated eye-head gaze shifts that obey Donders' laws of the eyes and head. The following features were specifically built into the model: (1) the model transforms eye-centered retinal inputs into eye and head rotations in head and shoulder-fixed coordinate systems, respectively, (2) the model applies experimentally observed behavioral constraints on the final orientations of eye (Listings law) and head (Fick strategy), and (3) variability in both eye-head contribution (including relative horizontal-vertical contributions) and influence of the VOR were implemented, without affecting the accuracy of the gaze shift or the spatial constraints named above. Our simulations show that the model was successful in realistically rendering each of these properties.

Two further novel and important properties emerged from our model simulations. First, without placing any additional dynamic constraints on the model, it predicted deviations in eye and head trajectories from the Listing and Fick between stable visual fixations that have been observed experimentally. Second, the model predicts that different patterns of eye-head coordination interact with the 3-D eye (Listing) and head (Fick) constraints to produce very different ranges of final eye-in-space orientations, with quite different consequences for vision.

Thus, our model provides both explanatory and predictive power for understanding known, and yet-to-be tested, aspects of 3-D gaze behavior. And as illustrated in Figure 1, our model provides a general framework for understanding the neural control system for the kinematics of head-free gaze control. Finally, the kinematic framework provided here provides a convenient stepping stone for further modeling studies of gaze dynamics and artificial neural network models that may further help to understand the neurophysiology of brain areas involved in gaze control.

## Funding

MD was supported by the Collaborative Research and Training Experience (CREATE) program of the National Science and Engineering Research Council (NSERC) of Canada. JC was supported by a Canada Research Chair Award.

### Conflict of interest statement

The authors declare that the research was conducted in the absence of any commercial or financial relationships that could be construed as a potential conflict of interest.
